# Supplementary material for: Bilingual Spatial Cognition: Spatial Cue Use in Bilinguals and Monolinguals
Source: Brain Sci. 2024 Jan 27;14(2):134. doi: 10.3390/brainsci14020134 (PMC10887090; doi:10.3390/brainsci14020134)
Supplement: Supplementary file 1 [file brainsci-14-00134-s001.zip › brainsci-2804010-supplementary.pdf]

Table S1. Reaction times on the Flanker Task per group and condition

	Mean	SD
Bilinguals		
Incongruent	457.54	42.88
Congruent	407.92	46.38
Neutral	408.86	45.26
Monolinguals		
Incongruent	545.66	73.92
Congruent	479.41	60.55
Neutral	475.04	61.18

Table S2. ENCODING. Brain regions showing significantly more activity for bilinguals than monolinguals (statistical threshold at p<.001 uncorrected).

Region	BA	Cluster Extent (voxels)	Peak Voxel Level		MNI coordinates		
			T	equiv Z	x	y	z
**EFFECT OF GROUP**							
**Bilinguals > Monolinguals**							
Right middle temporal gyrus	21	6919	6.28	5.35	46	-54	10
Right fusiform gyrus	18		6.18	5.29	24	-72	-14
Left fusiform gyrus	19		5.95	5.14	-28	-68	-6
Right inferior frontal gyrus (p. opercularis)	44	1150	5.46	4.80	58	14	30
Right cerebral white matter	48		5.26	4.66	40	2	22
Right precentral gyrus	6		4.87	4.38	58	6	38
Left inferior temporal gyrus	20	1084	5.40	4.76	-46	0	-38
Left inferior frontal gyrus (p. orbitalis)	38		5.11	4.55	-26	18	-24
Left temporal pole	38		5.06	4.52	-46	18	-22
Left supplementary motor area	8	814	5.31	4.69	-8	26	50
Right supplementary motor area	8		4.76	4.30	10	26	56
Left superior medial frontal gyrus	32		4.24	3.90	-8	38	38
Left inferior parietal cortex	39	726	5.48	4.82	-48	-58	44
Left supramarginal gyrus	40		4.41	4.03	-58	-46	36
Left angular gyrus	39		4.24	3.89	-44	-66	50
Left postcentral gyrus	4	324	5.05	4.51	-58	-2	40
Left precentral gyrus	4		4.74	4.28	-60	0	32
Left inferior frontal gyrus (p. triangularis)	44		4.65	4.21	-58	14	30
Left cerebral white matter	21	384	4.72	4.27	-46	-28	-2
Left middle temporal gyrus	21		4.17	3.84	-54	-34	-4
Left cerebral white matter	-		3.46	3.25	-40	-42	6
Right superior frontal gyrus	9	314	6.25	5.33	14	60	36
Right superior frontal gyrus	9		4.12	3.82	18	46	48
Right middle frontal gyrus	-		3.59	3.37	28	60	28
Right cerebral white matter	2	261	4.25	3.91	46	-30	36
Right supramarginal gyrus	2		4.24	3.90	58	-32	34
Right supramarginal gyrus	48		4.16	3.89	42	-30	26

Left supramarginal gyrus	2	198	4.69	4.24	-52	-28	36
Left supramarginal gyrus	2		4.14	3.82	-60	-24	40
Left cerebral white matter	48		3.96	3.67	-42	-30	34
**OBJECTS TRIALS**							
**Bilinguals > Monolinguals**							
Left inferior parietal cortex	40	531	4.63	4.20	-48	-54	42
Left inferior parietal cortex	40		3.76	3.51	-58	-46	36
Left inferior parietal cortex	40		3.74	3.49	-52	-46	42
Right angular gyrus	39	498	4.64	4.20	50	-66	42
Right middle occipital gyrus	39		4.29	3.93	38	-72	28
Right cerebral white matter	19		3.99	3.70	28	-64	22
Right middle temporal gyrus	21	397	5.35	4.72	46	-54	10
Right middle temporal gyrus	21		4.06	3.75	52	-46	4
Right middle temporal gyrus	37		3.73	3.49	58	-64	14
Right fusiform gyrus	18	239	4.41	4.03	24	-72	-14
Right cerebelum	18		4.14	3.82	16	-74	-16
Right fusiform gyrus	19		3.68	3.44	26	-72	-6
Left inferior temporal gyrus	20	202	4.70	4.25	-46	0	-38
Left cerebral white matter	20		3.47	3.27	-40	-6	-24
Left temporal pole	38		3.44	3.24	-34	10	-24
**SHADOW TRIALS**							
**Bilinguals > Monolinguals**							
Right lingual gyrus	18	1364	5.29	4.68	8	-74	-8
Left fusiform gyrus	19		5.29	4.68	-28	-72	-8
Right fusiform gyrus	18		5.27	4.66	24	-72	-14
Right calcarine gyrus	17	491	4.41	4.03	18	-58	10
Right calcarine gyrus	17		4.18	3.85	8	-64	12
Right cuneus	18		4.16	3.83	10	-82	18
Right cerebral white matter	19	409	4.57	4.15	36	-72	2
Right middle occipital gyrus	19		4.40	4.02	44	-82	10
Right middle occipital gyrus	39		4.34	3.97	38	-72	28
Left inferior frontal gyrus, (p. orbitalis)	38	407	5.23	4.64	-26	18	-24
Left inferior frontal gyrus, (p. orbitalis)	11		4.50	4.10	-20	12	-18
Left cerebral white matter	48		3.92	3.64	-38	6	-16
Right precentral gyrus	6	286	4.44	4.05	58	6	36
Right inferior frontal gyrus (p. opercularis)	44		4.14	3.82	58	14	28
Right precentral gyrus	6		3.63	3.40	50	8	48

Table S3. RETRIEVAL – CONGRUENT. Brain regions showing significantly more activity for bilinguals than monolinguals (statistical threshold at p<.001 uncorrected).

Region	BA	Cluster Extent (voxels)	Peak Voxel Level		MNI coordinates		
			T	equiv Z	x	y	z

**EFFECT OF GROUP**							
**Bilinguals > Monolinguals**							
Right superior frontal gyrus	10	508	6.15	5.27	16	62	24
Right middle frontal gyrus	46		5.38	4.75	26	62	26
Right superior medial frontal gyrus	9		4.82	4.34	10	60	38
Right cerebellum	30	390	6.21	5.31	24	-32	-24
Right fusiform gyrus	37		5.26	4.66	32	-34	-14
Right cerebral white matter	-		4.47	4.07	24	-18	-30
Left caudate nucleus	-	388	5.74	5.00	-6	16	10
Left superior orbitalfrontal gyrus	11		5.14	4.58	-22	20	-12
Left cerebral white matter	11		4.60	4.17	-18	24	-6
Right superior orbitalfrontal gyrus	11	339	7.13	5.88	16	56	-10
Right superior frontal gyrus	10		4.70	4.25	18	60	4
Right rectus	11		4.59	4.16	6	52	-18
Right rolandic operculum	48	289	6.87	5.72	54	-18	22
Right superior temporal gyrus	22		3.77	3.52	66	-12	12
Left superior temporal gyrus	48	267	6.54	5.52	-48	6	-12
Left superior temporal pole	38		4.90	4.40	-44	26	-18
Left cerebral white matter	-		4.77	4.30	-42	36	-20
Left parahippocampal gyrus*	37	101	5.26	4.66	32	-34	-14
			4.64	4.20	26	-28	-20
			4.53	4.12	24	-26	-24
			4.47	4.07	24	-18	-30
			4.27	3.92	26	-22	-28
			3.91	3.63	26	-30	-16
Right parahippocampal gyrus*	36	77	6.14	5.26	-22	-16	-32
			4.26	3.91	-22	-24	-26
Right hippocampus*	20	53	4.36	3.99	26	-14	-12
			3.57	3.35	40	-12	-14
**OBJECTS TRIALS**							
**Bilinguals > Monolinguals**							
Left caudate nucleus*	-	25	4.36	3.99	-6	16	8
**SHADOW TRIALS**							
**Bilinguals > Monolinguals**							
Right superior frontal gyrus	10	267	5.18	4.60	16	62	24
Right middle frontal gyrus	46		4.45	4.06	26	62	26
Right superior medial frontal gyrus	9		3.90	3.62	10	60	38
Left caudate nucleus*	-	20	4.07	3.76	-6	-16	8
		19	3.66	3.43	-16	24	-6
			3.58	3.36	-10	22	-6
Left parahippocampal gyrus*	36	35	4.97	4.45	-22	-16	-32

*p < .05 SVC

Table S4. RETREIVAL – INCONGRUENT. Brain regions showing significantly more activity for bilinguals than monolinguals (statistical threshold at p<.001 uncorrected).

Region	BA	Cluster Extent (voxels)	Peak Voxel Level		MNI coordinates		

			T	equiv Z	x	y	z
**EFFECT OF GROUP**							
**Bilinguals > Monolinguals**							
Right superior frontal gyrus	10	2339	6.93	5.76	18	58	18
Right inferior frontal gyrus (p. triangularis)	45		6.49	5.49	56	34	2
Right superior medial frontal gyrus	9		6.10	5.23	10	60	38
Right inferior temporal gyrus	20	1159	5.61	4.90	44	-12	-28
Right cerebral white matter	-		5.21	4.62	22	-14	-10
Right parahippocampal gyrus	37		5.14	4.57	34	-34	-14
Left cerebral white matter	-	726	5.63	4.92	-6	-12	30
Right superior frontal gyrus	6		5.46	4.80	14	14	54
Right cerebral white matter	-		4.44	4.05	6	8	28
Left caudate nucleus	25	461	5.10	4.54	-6	16	8
Right cerebral white matter	25		4.38	4.00	4	30	4
Left anterior cingulate cortex	11		4.14	3.81	-4	40	4
Right rolandic operculum	48	331	6.96	5.78	52	-18	24
Right supramarginal gyrus	48		4.40	4.02	60	-26	28
Right supramarginal gyrus	2		4.36	3.99	62	-24	36
Left cerebral white matter	48	248	4.62	4.19	-24	20	-12
Left inferior orbital frontal gyrus	47		4.40	4.02	-34	26	-14
Left superior temporal pole	38		4.07	3.76	-44	26	-18
Right hippocampus*	-	105	4.47	4.08	24	-14	-12
			4.23	3.89	40	-12	-24
			3.56	3.34	42	-16	-18
			3.49	3.28	30	-12	-26
			3.47	3.27	40	-22	-18
**OBJECTS INCONGRUENT TRIALS**							
**Bilinguals > Monolinguals**							
Right superior frontal gyrus	10	273	4.92	4.42	18	58	18
Right superior medial frontal gyrus	9		4.17	3.84	10	60	38
Right cerebral white matter	-		3.76	3.50	20	46	22
Left caudate nucleus*	-	26	4.13	3.80	-6	16	8
			3.63	3.40	-4	18	2
**SHADOW INCONGRUENT TRIALS**							
**Bilinguals > Monolinguals**							
Right superior frontal gyrus	10	1221	5.83	5.05	18	58	18
Right superior medial frontal gyrus	10		5.50	4.83	14	60	2
Right superior medial frontal gyrus	9		5.29	4.69	10	60	38
Right inferior temporal gyrus	20	263	4.76	4.30	44	-12	-30
Right inferior temporal gyrus	20		3.92	3.64	50	-6	-36
Right cerebral white matter	36		3.48	3.28	34	4	-30

Right hippocampus*	-	44	4.54	4.13	24	-14	-12
Left caudate nucleus*	-	21	3.78	3.52	-6	16	8
			3.70	3.45	-4	20	0
Left parahippocampal gyrus*	-	55	4.58	4.16	-24	-18	-30

*p < .05 SVC
